# 4022 Recruiting hidden and sensitive populations: methods for recruitment of pregnant women who regularly use cannabinoids

**DOI:** 10.1017/cts.2020.142

**Published:** 2020-07-29

**Authors:** Stefanie Kennon-McGill, Lindsey Sward, Clare Nesmith, Laura P James

**Affiliations:** 1 University of Arkansas Translational Research Institute; 2 University of Arkansas for Medical Sciences

## Abstract

OBJECTIVES/GOALS: Prenatal cannabinoid use is increasing and more studies are needed to describe the neurodevelopmental impact on the fetus. However, pregnant cannabinoid users are a “hidden population,” which makes identification of these individuals for research difficult. Our study will employ three methods of recruitment and evaluate the success of each method. METHODS/STUDY POPULATION: We will recruit a total of 40 women in the third trimester of pregnancy who regularly use cannabinoid products thought to contain tetrahydrocannabinol (THC) and/or cannabidiol (CBD) throughout their pregnancies, and 20 control pregnant women who do not use those products. The purpose of this study is to evaluate the effects of prenatal cannabinoid use on the neurodevelopment of their offspring over the first year of life. We will employ three recruitment methods. First, targeted recruitment will occur in two university-based obstetrical clinics, where the obstetrician will present the study material and contact information. Second, we will utilize social media advertisements targeted to a specific demographic of Facebook users. Finally, we will employ the traditional method of distributing flyers in a non-targeted manner. We will track methods of recruitment success and gather information from the mothers on their preferences for recruitment approaches. RESULTS/ANTICIPATED RESULTS: Recruitment will start in January 2020 and continue for several months. We anticipate that the targeted method will yield the highest number of participants, and participants with the best fit for the inclusion criteria. However, it is possible that those women will be deterred by fear of having their drug use status revealed to their care providers, even though all research activity will occur independently from clinic visits and will not be transmitted to the electronic health record. The inclusion of a control group will also help foster “anonymity” for participants. The social media method has the potential for the greatest reach, but we expect many of these potential participants will fail to meet inclusion/exclusion criteria, as this is not as targeted as the first method. We anticipate a similar issue with the flyer-based approach. DISCUSSION/SIGNIFICANCE OF IMPACT: Optimizing recruitment of hidden and sensitive populations is crucial for clinical and translational research. Our goal is to identify strategies that can lead to best practices for engagement of those populations. Our conclusions could be applied in recruitment of sensitive populations for other clinical and translational research projects.

